# Wandel von Nutzen und Risiko antidepressiver Pharmakotherapie

**DOI:** 10.1007/s00115-024-01672-y

**Published:** 2024-05-16

**Authors:** Christopher Baethge

**Affiliations:** Klinik für Psychiatrie und Psychotherapie, Universität zu Köln, Kerpener Straße 62, 50937 Köln, Deutschland

**Keywords:** Effektstärke, Suizidprävention, Absetzsymptom, Rebound-Phänomen, Psychiatrie, Treatment effectiveness, Suicide prevention, Withdrawal symptom, Rebound phenomenon, Psychiatry

## Abstract

Die antidepressive Pharmakotherapie durchlief in ihrer Geschichte verschiedene Phasen: Der Euphorie der Anfangsjahre über die medikamentöse Erleichterung depressiver Syndrome folgte ein langer Zeitraum klinischer Erfahrung und intensiver wissenschaftlicher Durchdringung, die zu einer abgewogeneren Perspektive führten. Aktuelle Debatten kreisen um die tatsächliche Effektstärke – gerade in Bezug auf lange Behandlungsdauern –, die Prävention von Suiziden und die Folgen des Absetzens eines Antidepressivums. Die Bewertung der Stoffgruppe, aber oft auch das Nutzen-Schaden-Verhältnis einer individuellen Behandlung verändert sich mit der Zeit. Die Antidepressiva stehen exemplarisch für viele psychiatrische Behandlungen, die – in einem Begriff Hanfried Helmchens – ebenso janusköpfig sind wie die Psychiatrie es als Wissenschaft und als klinisches Fach ganz allgemein ist.

## Abstract (en)

Antidepressive pharmacotherapy has undergone various phases in its history. The euphoria of the early years on the relief of depressive symptoms was followed by a long period of clinical experience and intensive scientific work resulting in a more balanced perspective. Current debates circle around the actual effectiveness, especially with respect to long-term treatment, the prevention of suicide and the sequelae of discontinuation of an antidepressant. The evaluation of antidepressants as a group and often also the risk-benefit ratio of an individual treatment change over time. Antidepressants are typical for many forms of psychiatric treatment which, in a term from Hanfried Helmchen, are just as Janus-faced as psychiatry in a general sense is as a science and as a clinical discipline.

„… Suche nach Gemeinsamkeiten in den verschiedenen Blicken auf die Realität, Anerkennung, dass die Realität verschiedene Facetten hat und verschiedene Blicke darauf möglich sind, vielleicht sogar den eigenen Blick durch Berücksichtigung des anderen … zu erweitern …, ist Ziel dieses Buches.“ (Helmchen [1])

In *Das Janusgesicht der Psychiatrie*, dem das obige Zitat entnommen ist, werden die vielen Ambivalenzen der Psychiatrie angesprochen: etwa die Spannung zwischen Partizipation und Paternalismus und die damit verwandte zwischen Zwang und Freiwilligkeit, aber auch der Konflikt eines primär biologischen psychiatrischen Weltbildes mit einem eher sozialen oder psychologischen und nicht zuletzt die Balance von Gewinn und Gefahren der Behandlungen. Diese Spannungen sind nach Helmchen nicht aufzulösen, und er zieht daraus die Konsequenz, sie anzuerkennen und nutzbar zu machen. Und so beschreibt das zunächst fast trivial wirkende Zitat nicht nur ein wissenschaftliches, ein intellektuelles Programm, sondern auch eine ärztliche und menschliche Haltung, über die nachzudenken es sich lohnt. Im Folgenden geschieht dies am Beispiel des Nutzens und Risikos der Pharmakotherapie mit Antidepressiva – einfach, weil sie so viele Menschen betrifft, und überdies weil der Verfasser auf diesem Gebiet klinische und wissenschaftliche Erfahrungen gesammelt hat.

## Nutzen und Risiken von Antidepressiva – die Perspektive hat sich geändert

### Nutzen

Gegenwärtig erzeugt die antidepressive Wirkung von Psilocybin Enthusiasmus, und vielleicht ähnelt die Situation der Zeit der ersten Antidepressivastudien in den 1950er-Jahren, deren Ergebnisse auf eine sehr starke Wirksamkeit von Imipramin hindeuteten [2]. Heute hat eine kritischere Einschätzung – nicht insbesondere der trizyklischen, sondern aller antidepressiven Wirkstoffe –, z. T. sogar ein gewisser Nihilismus, diesen Optimismus ersetzt: Die Remissionsrate unter Behandlung mit Antidepressiva beträgt nur etwa ein Drittel [3], und mittlerweile scheitert ungefähr die Hälfte aller randomisierten kontrollierten Studien (RCT) zum Thema Antidepressiva [4]. Zumindest gemessen an den durch Cohen etablierten Konventionen von Effektstärken in der Medizin scheint sich über alle randomisierten Studien mittlerweile eine moderate Wirksamkeit herauszumitteln, die gegenüber Placebo etwa 0,3 standardisierten Mittelwertsunterschieden entspricht [5].

Ähnlich verhält es sich mit der antisuizidalen Wirkung: Antidepressiva helfen gegen Selbstmordgedanken und -absichten [6, 7], was die Annahme einer prophylaktischen Wirkung auf vollendete Suizide nahelegt. In diesem Sinne haben Beobachtungsstudien eine Assoziation von steigenden Verkaufszahlen der selektiven Serotonin-Wiederaufnahmehemmer („selective serotonin reuptake inhibitor“, SSRI) und abnehmenden Suizidraten gezeigt (etwa: [8, 9]). Diese Assoziation ist jedoch umstritten, denn eine aktuelle systematische Aufarbeitung und Metaanalyse von Beobachtungsstudien hat sie nicht bestätigen können [10]. Auch die in den vergangenen zwei Jahrzehnten durchgeführten Metaanalysen randomisierter Studien konnten eine Reduzierung des Risikos von Suiziden nicht belegen (etwa [11–13]).

Antidepressive und suizidverhindernde Wirkungen: Folgt man den vielen Vergleichen mit Placebo, hat sich seit dem Beginn der psychopharmakologischen Ära vor 70 Jahren der Nutzen der Antidepressiva nach unten relativiert.

Diese Eintrübung anfänglicher Begeisterung ist weder auf die Antidepressiva beschränkt, noch ist sie eine Eigenschaft psychiatrischer Therapien. Das „Proteus-Phänomen“ von zunächst starken und später abgeschwächten Effekten hat sich in klinischen Studien auch auf anderen Gebieten, besonders aber in der Grundlagenforschung gezeigt [14]. Ein möglicher Mechanismus wäre das Zusammenspiel von a) der Anfälligkeit oft kleiner erster Studien für Ausreißerergebnisse und starke Effekte mit b) der Suche nach publikationsfördernden statistisch signifikanten Ergebnissen, die sich bei großen Wirkstärken eher ergeben. Ein anderer Grund könnte in der Indikationserweiterung für eine einmal als wirksam etablierte Therapie bestehen. In den ersten Imipraminstudien etwa hatten Patientinnen und Patienten mit endogener Depression am besten respondiert [15], ohne dass sich die Verschreibung in der Folgezeit auf diesen Subtyp beschränkt hätte. Nicht zuletzt könnte die umfassende Betreuung der Patientinnen und Patienten in den Placebogruppen der RCT den Kontrast zu den Verumgruppen abgeschwächt haben. Zudem zeigen sich bei depressiven Störungen oft auch unter Placebo oft recht gute Verläufe, zumindest im Vergleich mit anderen psychischen Störungen [16].

### Risiken

Die Perspektive auf die Nachteile der Medikamententherapie von Patientinnen und Patienten mit depressiven Störungen hat sich in der Wissenschaft über die letzten Jahrzehnte ebenfalls geändert. Ein Beispiel ist die Diskussion über die Absetzsymptome („antidepressant discontinuation symptoms/syndromes“, ADS). Während Andersen und Kristiansen Absetzsymptome bei Imipramin bereits 1959 beschrieben haben [17], standen sie lange nicht im Vordergrund, auch nicht in den Leitlinien [18]. In den letzten Jahren, beginnend mit einer Übersichtsarbeit von Davies und Read [19] und ihrer Aufnahme in die Laienpresse, etwa im meinungsbildenden *New Yorker*, rückten ADS ins Zentrum der Debatte über Antidepressiva. Heute scheint sich abzuzeichnen, dass eine signifikante Minderheit nach Beendigung einer antidepressiven Medikamentenbehandlung ein genuines Absetzsymptom erlebt, und dass schwere Bilder relativ selten zu sein scheinen [20]. Unter dem Gesichtspunkt der Veränderung von Vor- und Nachteilen der Behandlung über die Zeit ist auffällig, dass eine längere Antidepressivatherapie ein Risikofaktor für das Auftreten von ADS sein könnte [20].

Weit weniger geklärt und in ihren möglichen Auswirkungen bedrohlicher ist die Frage nach einer erhöhten Anfälligkeit für depressive Erkrankungen nach einer Medikamentenbehandlung im Sinne eines Rebound [21]. Hintergrund ist die paradoxe Wahrnehmung, nach der die Verschreibung von Antidepressiva anhaltend zunimmt (etwa [22]), die Häufigkeit depressiver Erkrankungen aber nicht zurückgeht (z. B. [23]). Eine Erklärungshypothese dieses Widerspruchs geht von der Förderung der serotonergen und noradrenergen Transmission durch die Substanzen aus, die zu einer kompensatorischen Gegenregulation führt, mit der Folge eines funktionellen Serotonin- und Noradrenalinmangels nach Absetzen und einer erhöhten Wahrscheinlichkeit für das Auftreten einer depressiven Symptomatik. Während das epidemiologische Paradox sicher besteht und einer Erklärung bedarf, soll auch darauf hingewiesen werden, dass das pathophysiologische Modell oder überhaupt eine Rolle der Antidepressiva bei der Entstehung depressiver Erkrankungen bisher nicht gesichert ist.

Zumindest hat sich in der jüngeren Vergangenheit auch mit Blick auf die Schadwirkungen eine Perspektivverschiebung ergeben: Im Vergleich zu ihrer Einführung in den 1950er- und 1960er-Jahren oder auch zur Zeit ihrer Erneuerung, wenn man die Entdeckung der SSRI vor 35 Jahren so versteht, sind die Risiken heutzutage stärker im Bewusstsein. Sieht man von der Phase einer pauschalen Ablehnung der Psychopharmaka in den 1970er- und 1980er-Jahren ab, kommt die heutige Psychiatrie zu einer kritischeren Beurteilung der Antidepressiva als frühere Psychiaterinnen und Psychiater.

### Unsicherheiten während einer Behandlung

Aber auch über die Strecke einer individuellen Behandlung mit einem Antidepressivum verändert sich die Bewertung. Die Ergebnisse für die Akutbehandlung über einige Wochen und Monate sind robust [24], aber je länger in der Akuttherapie auf einen Erfolg gewartet werden muss, desto geringer ist die wissenschaftliche Basis für die Wirksamkeit der Behandlung, sodass – wenn auch nicht unbedingt die Effektivität –, so doch die Gewissheit über den Gewinn für die Betroffenen sinkt.

Unklarheit besteht zudem über den natürlichen Verlauf: Während genug Erfahrungen zu den ersten Wochen einer depressiven Störung vorliegen, ist der unbehandelte Verlauf über mehrere Monate weitgehend unbekannt, einfach weil *irgendeine* Form der Therapie seit Langem üblich ist. Zumindest konnten Whiteford et al. [25] in einer systematischen Übersicht nur sehr unbestimmte Schätzungen dazu präsentieren, mit welcher Wahrscheinlichkeit eine Remission nach einem Viertel- oder einem halben Jahr eintreten könnte (rund ein Viertel nach 3, ein Drittel nach 6 und die Hälfte nach 12 Monaten). Diese Wissenslücke wird sich so bald nicht schließen, sodass mit zunehmender Dauer der Erkrankung an die Seite der Unsicherheit über den therapeutischen Effekt mangelndes Wissen über den anzunehmenden Fortgang der unbehandelten Erkrankung tritt. Beides verändert die Aufklärung der Patientinnen und Patienten, und beides führt zu mehr Unschärfe in der individuellen Nutzen-Risiko-Bilanz, je länger die Behandlung dauert.

## Der Januskopf

Die Beispiele sollen zeigen, was mit einer Veränderung des Nutzen-Risiko-Verhältnisses über die Zeit in der Psychiatrie gemeint ist (Helmchen [1]). Gleichzeitig besteht auch im persönlichen Krankheitsverlauf eine solche Verschiebung, weil wir über den Nutzen eines Medikaments bei langfristiger Therapie mindestens weniger wissen als zu Beginn und vielleicht sogar eine Zunahme der Schadwirkungen im Verlauf befürchten müssen, wie die Beispiele ADS und Rebound, aber auch der unerwünschten Arzneimittelwirkungen (UAW wie Gewichtszunahme, Frakturen und sexuelle Nebenwirkungen) zeigen. Das Bild vom Januskopf aufnehmend, könnte man sagen, er wendet uns mit der Zeit – und hier ist der Verlauf einzelner an Depressionen leidender Menschen genauso gemeint wie die Geschichte der Wirkungen von Antidepressiva –, etwas mehr sein ernüchtertes Haupt zu, während wir von seinem begeisterten Antlitz weniger sehen.

Im Ausschnitthaften der Beispiele können die Fortschritte aus dem Blick geraten: Tatsächlich steht mit Lithium ein psychopharmakologisches Prinzip zur Suizidprophylaxe zur Verfügung [26], und es existiert eine Fülle effektiver Behandlungen depressiver Erkrankungen: die Antidepressiva selbst und ihre Kombinationen [27], andere antidepressiv wirkende Substanzen, die Hirnstimulationsverfahren sowie allgemeine und störungsspezifische Psychotherapien [28]. Auch ist das Niveau der wissenschaftlichen Aufarbeitung gestiegen, was an den RCT selbst, aber auch an der Leitlinienkultur, konkret an der Nationalen VersorgungsLeitlinie Unipolare Depression [28] nachzuvollziehen ist. Nicht zuletzt haben psychische Erkrankungen einen Teil ihres Stigmas verloren.

Umgekehrt sind die Betroffenen selbstbewusster und emanzipierter als früher, und sie können Informationen über die Vor- und Nachteile der Behandlungen erwarten. Wie gezeigt, bedeutet dies, gerade etwas über Ungewissheiten zu erfahren, über eine umso unklarere Balance von Kosten und Nutzen, je länger die Erkrankung und ihre Behandlung dauern – ein Anspruch an Aufklärung, der auch mit der depressionstypischen und manchmal selbstzerstörerischen Neigung zu Zweifeln und Pessimismus in Konflikt treten kann.

## Die Tradition Griesingers

Das Leitmotiv des Januskopfes [1] und der Vielschichtigkeit ist nützlich für die Diskussion über das Für und Wider der antidepressiven Pharmakotherapie, in seinen Spielarten des Abwägens von Gewinn und Gefahr oder von Aufwand und Ausbeute gilt es jedoch auch darüber hinaus: Von Wilhelm Griesinger (1817-1868) ist das Axiom bekannt, nach dem alle Geisteskrankheiten Gehirnkrankheiten seien. Genau besehen, hat er es so formuliert:

„Welches Organ muss also überall und immer nothwendig erkrankt sein, wo Irresein vorhanden ist? — Die Antwort auf diese Frage ist die erste Voraussetzung der ganzen Psychiatrie.

Zeigen uns physiologische und pathologische Thatsachen, dass dieses Organ nur das Gehirn sein kann, so haben wir vor Allem in den psychischen Krankheiten jedesmal Erkrankungen des Gehirns zu erkennen.“ (Griesinger [29])

Gerade dem Reformer Griesinger kann kaum der Vorwurf eines primitiven Biologismus gemacht werden. Dennoch bestand lange, und besteht vereinzelt vermutlich immer noch, ein Zweig der Psychiatrie, den man eher biologistisch als biologisch nennen muss, zumindest insoweit er ungeachtet aller Fehschläge an einem exklusiv biologischen Paradigma festhält und sich Hoffnung auf klinische Verbesserungen primär von den Neurowissenschaften, namentlich von funktioneller Bildgebung und Genetik, verspricht. Unabhängig von der grundsätzlichen Plausibilität einer biologischen Komponente vieler psychischer Störungen und paradigmatischer Erkrankungen, wie etwa der Neurolues und der Demenzen, stellt sich die Frage nach der Erfolgsbilanz des obigen Diktums für die klinische Praxis, nach dem Verhältnis von Kosten und Nutzen einer biologistischen Psychiatrie. Helmchen schreibt nüchtern:

„Die biologischen Untersuchungsverfahren haben unser neurowissenschaftlich begründetes Wissen erheblich vermehrt und differenziert; allerdings hat dieses Wissen bisher relativ wenig praktischen Nutzen für die Behandlung von Patienten gebracht ….“ (Helmchen [1])

Jede und jeder, die mit an schweren psychischen Erkrankungen leidenden Menschen arbeiten, müssen sich den Erfolg eines primär neurowissenschaftlichen Paradigmas in der Psychiatrie wünschen. Er wäre eine Erlösung. Nur: Trotz aller Brillanz, trotz allen Schweißes und allen Geldes ist der Ertrag, nicht für die Neurowissenschaft, aber für die Versorgung gering geblieben – 179 Jahre nach Griesinger. Die wesentlichen biologischen und medikamentösen Therapien waren weitgehend Resultate aufmerksamer klinischer Beobachtungen und entstanden gerade nicht aus hypothesengeleiteter biologischer Forschung [30]. Entfernt erinnert das unverdrossene Hoffen auf die Neurowissenschaft zur Linderung des heutigen Leids an den Ruf nach technischen Lösungen der Klimakrise: So großartig das wäre, sollten wir uns allein auf ein solches Paradigma verlassen?

Diese Bilanz sollte gerade nicht in einen alten Lagerkampf zurückführen, etwa den zwischen Sozialpsychiatrie und biologischer Psychiatrie [31] oder den noch älteren zwischen Psychikern und Somatikern [32]. Derlei Auseinandersetzungen – ein Beispiel auf einem anderen Feld wäre die Kluft zwischen tiefenpsychologisch orientierter und kognitiv-behavioraler Psychotherapie – sind fruchtlos, weil sie zu wenig bestimmt sind von Sachauseindandersetzungen und zu sehr von Identitätsfragen.

Identität und Haltung, Emotionalität und Bekenntnis aber stehen am Beginn des 21. Jh. hoch im Kurs. Helmchen stellt dem einen sachlichen Aufruf zur Akzeptanz der Unterschiede und zum Versuch einer Synthese von Widersprüchen gegenüber. Der Weg dahin kann nur in einer offenen Diskussion bestehen. Aber auf den Leserbriefseiten psychiatrischer Zeitschriften – als einem Forum fachlicher Auseinandersetzungen – ist ein Rückgang wissenschaftlicher Diskussionsbeiträge festzustellen: Ausweislich einer PubMed-Abfrage im Sommer 2023 ging etwa die Zahl der *Letters to the Editor* im *American Journal of Psychiatry* von 286 zu ihrem Höhepunkt im Jahr 1986 auf 13 in 2022 zurück. Im *British Journal of Psychiatry* erschienen Anfang der 1990er-Jahre fast 200 Leserbriefe jährlich, während es zuletzt noch 5 waren. Das *Deutsche Ärzteblatt* registriert ebenfalls, wenn auch noch auf hohem Niveau, eine Abnahme wissenschaftlicher Diskussionsbeiträge. Dennoch scheint ein interessierter und fairer, aber in der Sache auch tatsächlich kritischer Austausch am ehesten zu einer Integration der Ambivalenzen der Psychiatrie und zu einer produktiven Koexistenz ihrer verschiedenen Weltbilder im Sinne Helmchens führen zu können.

## Fazit für die Praxis

- Die antidepressive Pharmakotherapie steht exemplarisch für viele psychiatrische Behandlungen, die – in einem Begriff Hanfried Helmchens – ebenso janusköpfig und ambivalent sind wie die Psychiatrie es als Wissenschaft und als klinisches Fach ganz allgemein ist.
- Die damit einhergehenden Spannungen sind nach Helmchen nicht aufzulösen, und dieser zieht daraus die Konsequenz, sie anzuerkennen und nutzbar zu machen.
- Der Weg dahin kann nur in einer offenen Diskussion bestehen. Ein interessierter und fairer, aber in der Sache kritischer Austausch scheint am ehesten zu einer Integration der Ambivalenzen der Psychiatrie und zu einer produktiven Koexistenz ihrer verschiedenen Weltbilder im Sinne Helmchens führen zu können.
